# Supplementary figures and images for: Seasonal variation in species composition, deltamethrin susceptibility, and kdr mutations in anopheles mosquitoes in Northwest Ethiopia
Source: PLoS One. 2026 Jun 5;21(6):e0350942. doi: 10.1371/journal.pone.0350942 (PMC13240861; doi:10.1371/journal.pone.0350942)

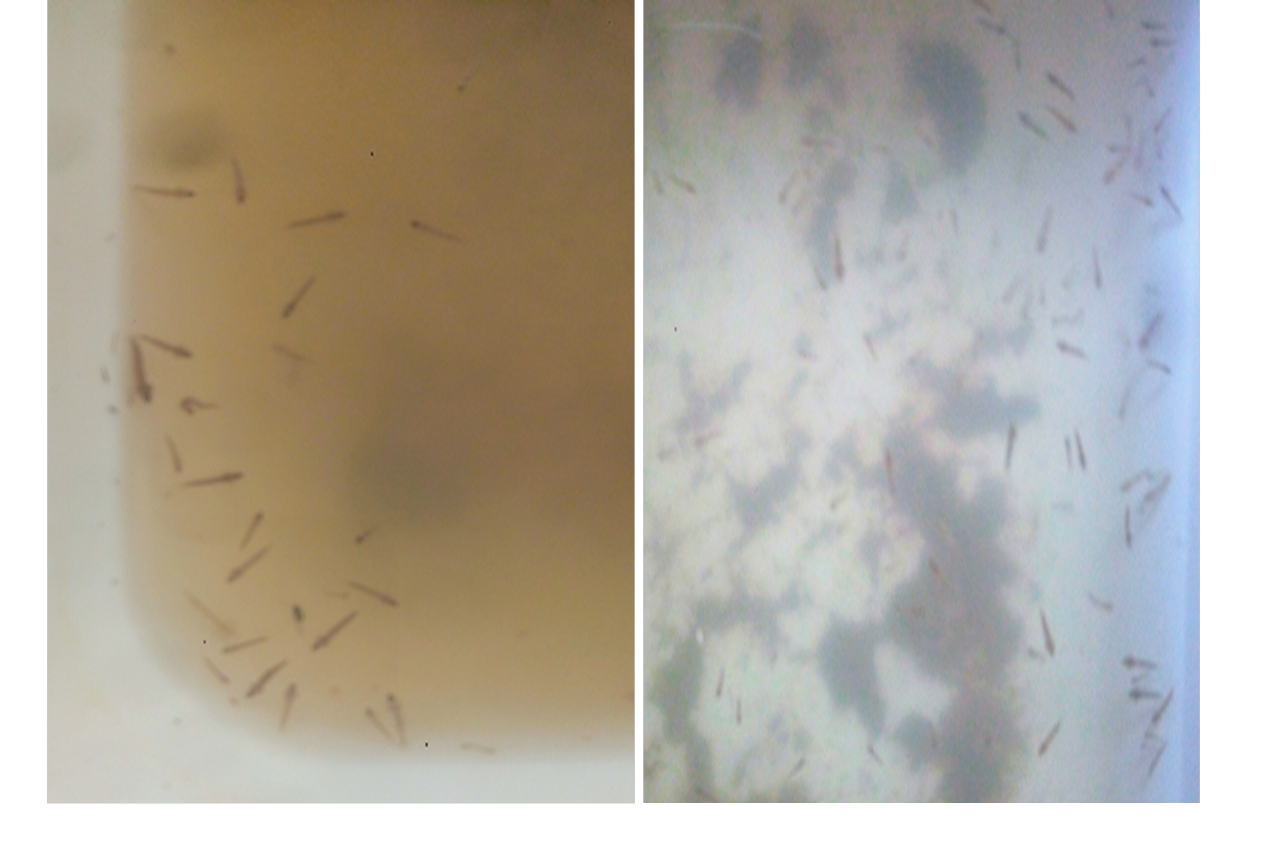

Supplement: S1 Fig — (TIF) [file pone.0350942.s001.tif]

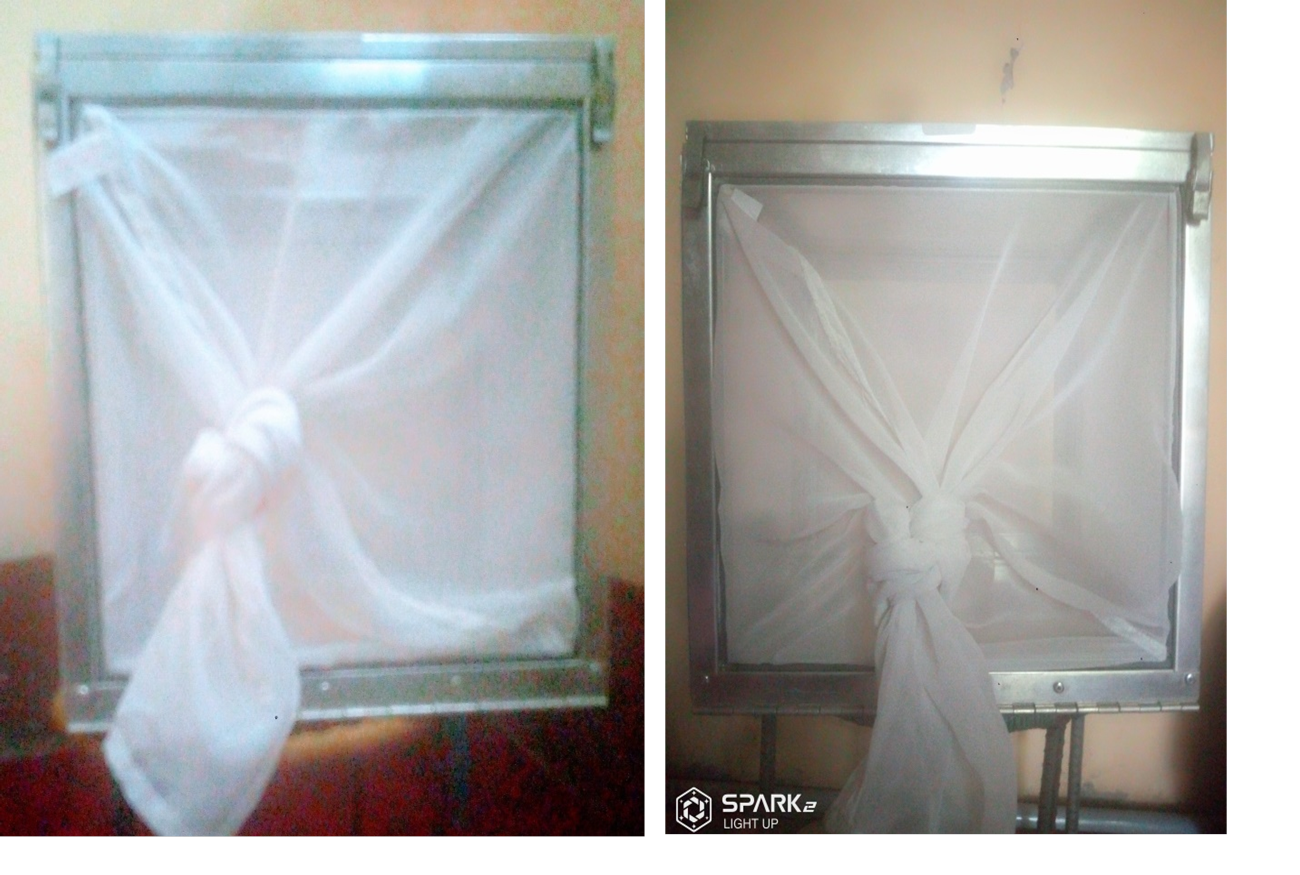

Supplement: S2 Fig — The figure represent the final stages of the rearing process, where field-collected pupae transition into adults within controlled cages. (TIF) [file pone.0350942.s002.tif]

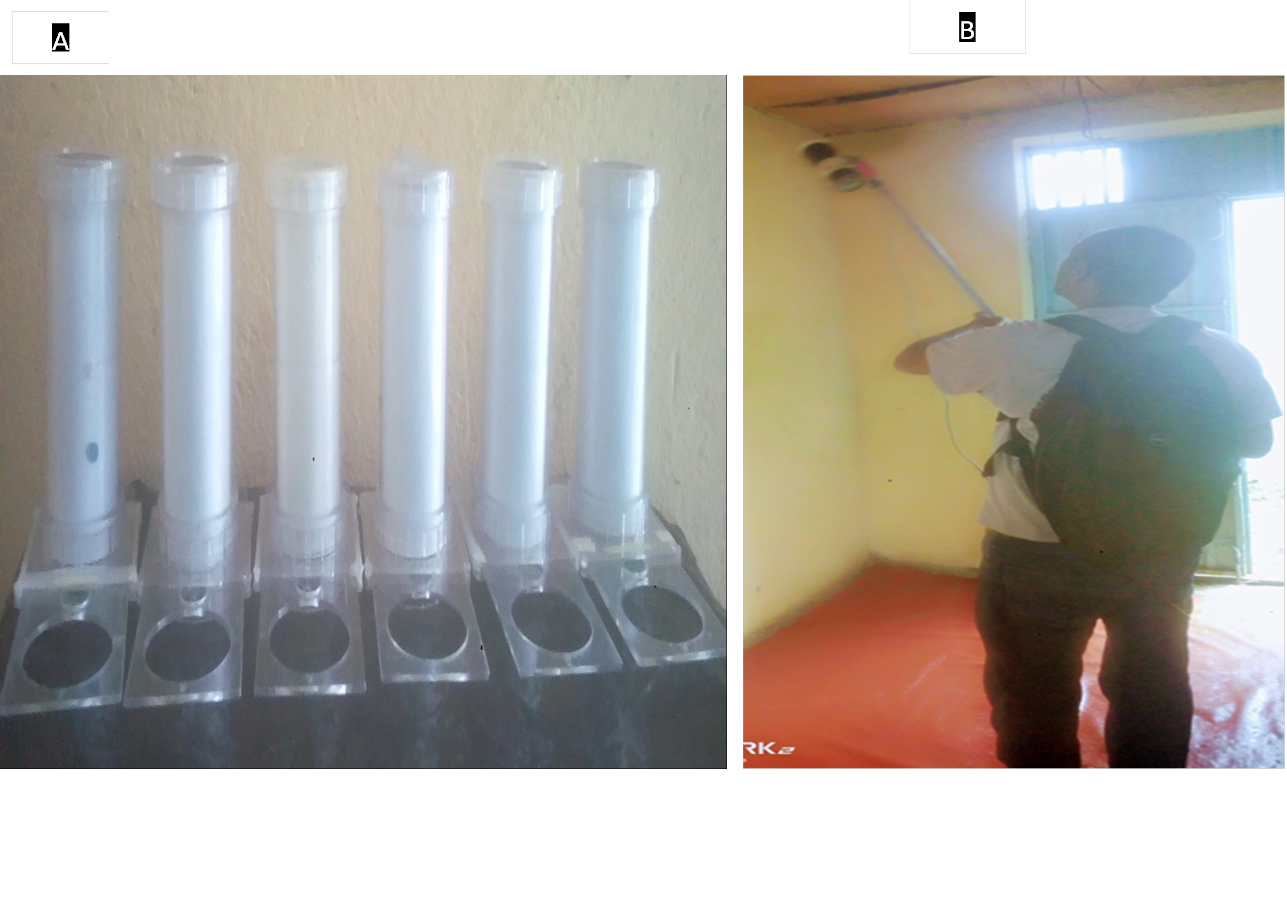

Supplement: S3 Fig — A: Displays the WHO susceptibility bio-assay tubes used to assess phenotypic resistance. B: Shows indoor adult female collection using a Prokopack aspirator in houses near larval breeding sites. (TIF) [file pone.0350942.s003.tif]

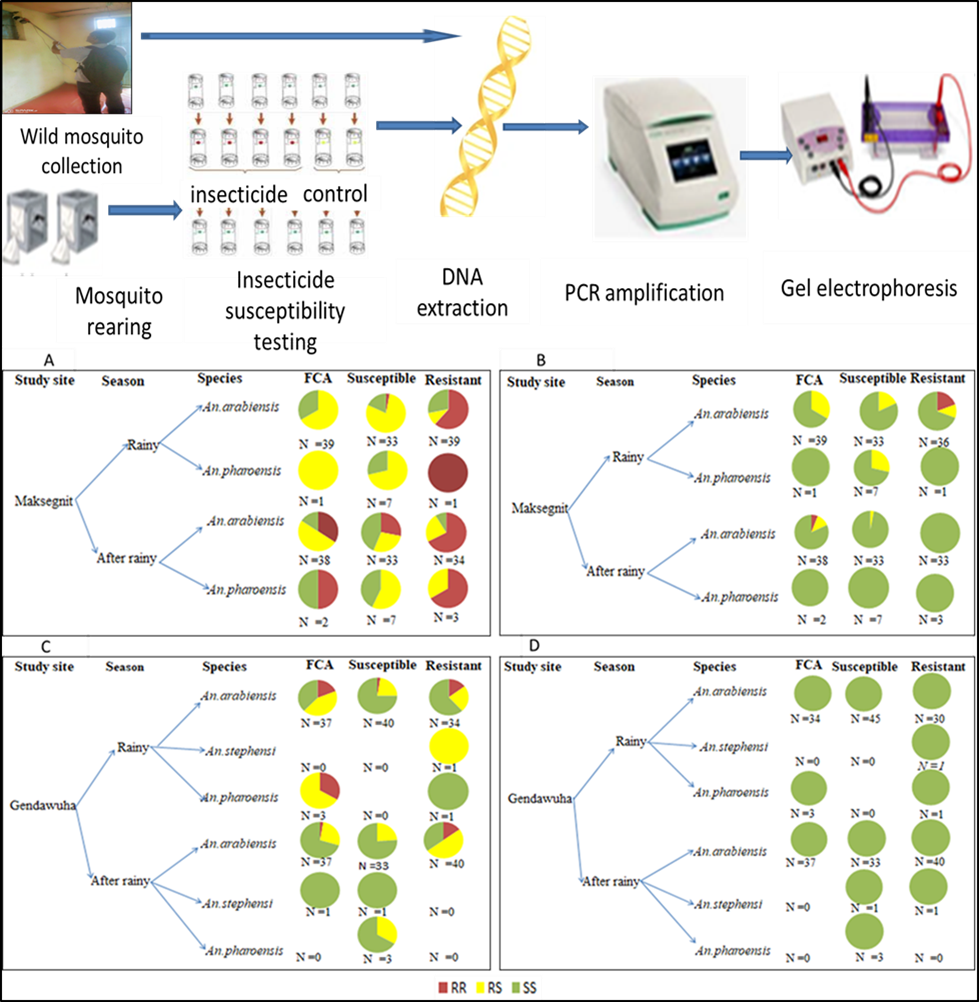

Supplement: S4 Fig — (TIF) [file pone.0350942.s004.tif]
