# Supplementary material for: Seasonal variation in species composition, deltamethrin susceptibility, and kdr mutations in anopheles mosquitoes in Northwest Ethiopia
Source: PLoS One. 2026 Jun 5;21(6):e0350942. doi: 10.1371/journal.pone.0350942 (PMC13240861; doi:10.1371/journal.pone.0350942)
Supplement: Attachment — Submitted filename: Response to reviewers.docx [file pone.0350942.s009.docx]

# May 9, 2026

# Response to Editors and Reviewers for PLOS one Submission

# PONE-D-26-04595R1

**Dear Editor and Reviewers,**

We are pleased to submit the revised version of our manuscript entitled “**Seasonal Variation in Species Composition, Deltamethrin Susceptibility, and kdr Mutations in Anopheles Mosquitoes in Northwest Ethiopia**”. We sincerely thank you for the insightful comments and constructive suggestions, which have greatly improved the quality of our work. Each point has been carefully considered, and revisions have been made accordingly. A point-by-point response to all comments is provided in the accompanying document. For clarity, responses are highlighted in blue with line numbers shaded in yellow referencing the corresponding revisions in the clean version of the manuscript.

We hope the revisions satisfactorily address all concerns, and we remain happy to provide any further clarifications if needed. We look forward to your kind consideration as the editorial process moves forward.

In this submission, we have included the following documents:

1. Revised manuscript with track changes
2. Revised clean manuscript
3. Main figures in separate files for individual figures
4. Supporting files
5. Supporting information files including row data, values used to build each pie chart and graph

Thank you once again for your thoughtful guidance and support in improving our study.

Sincerely,

Ligabaw Worku (on behalf of all co-authors)

Department of Medical Parasitology, College of Medicine and Health Sciences, University of Gondar, Po Box 196, Gondar, Ethiopia.

Email: [ligabaw@gmail.com](mailto:ligabaw@gmail.com)

# Point by point response to Editors

# Comment 1

# Please ensure that your manuscript meets PLOS ONE's style requirements, including those for file naming. The PLOS ONE style templates can be found at https://journals.plos.org/plosone/s/file?id=wjVg/PLOSOne_formatting_sample_main_body.pdf and

# https://journals.plos.org/plosone/s/file?id=ba62/PLOSOne_formatting_sample_title_authors_affiliations.pdf

**Response**

We have revised the manuscript to fully comply with PLOS ONE formatting guidelines, including file naming, in accordance with the provided templates.

**Comment 2**

In your Methods section, please provide additional information regarding the permits you obtained for the work. Please ensure you have included the full name of the authority that approved the field site access and, if no permits were required, a brief statement explaining why.

**Response**

We have revised the Methods section to include detailed information regarding the permits obtained for this study. Specifically, we have now clearly stated the full name of the authority that approved access to the field sites (line 118-119).

**Comment 3**

Your ethics statement should only appear in the Methods section of your manuscript. If your ethics statement is written in any section besides the Methods, please move it to the Methods section and delete it from any other section. Please ensure that your ethics statement is included in your manuscript, as the ethics statement entered into the online submission form will not be published alongside your manuscript.

**Response**

The ethics statement appears now exclusively in the Methods section, in line with PLOS ONE requirement (line 116).

**Comment 4**

We note that Figure 1 in your submission contain map images which may be copyrighted. All PLOS content is published under the Creative Commons Attribution License (CC BY 4.0), which means that the manuscript, images, and Supporting Information files will be freely available online, and any third party is permitted to access, download, copy, distribute, and use these materials in any way, even commercially, with proper attribution. For these reasons, we cannot publish previously copyrighted maps or satellite images created using proprietary data, such as Google software (Google Maps, Street View, and Earth). For more information, see our copyright guidelines: <http://journals.plos.org/plosone/s/licenses-and-copyright>.

**Response**

In response, we have removed the previously used map images and replaced Figure 1 with a newly generated map that complies with PLOS ONE’s copyright requirements and the Creative Commons Attribution License (CC BY 4.0). The original figure was prepared using a shapefile obtained from Africa GeoPortal, which requires written permission for publication. To address this, we regenerated the figure using a GADM GeoJSON dataset, which is freely available for academic use (see the new figure submitted separately).

**Comment 5**

We note that Figures S2, S3 and S4 in your submission contain copyrighted images. All PLOS content is published under the Creative Commons Attribution License (CC BY 4.0), which means that the manuscript, images, and Supporting Information files will be freely available online, and any third party is permitted to access, download, copy, distribute, and use these materials in any way, even commercially, with proper attribution. For more information, see our copyright guidelines: <http://journals.plos.org/plosone/s/licenses-and-copyright>.

**Response**

We would like to formally state that Figures S2, S3, and S4 are original photographs taken by the authors during the course of our study.

Specifically:

- Figure S2 (developing larvae): This image was captured by the authors during the mosquito rearing process to document larval development.
- Figure S3 (experimental setup for pupa emerging into adult): This image was captured by the authors in our field insectaries and laboratory facilities to show the setup for emerging adults.
- Figure S4 (susceptibility testing using WHO tube and indoor adult mosquito collection): These images were taken by the research team during the indoor adult mosquito collection process and subsequent phenotypic insecticide resistance testing in the field.

As the sole creators and owners of these images, we confirm that no third-party copyrights are involved. We fully agree to have these materials published under the Creative Commons Attribution License (CC BY 4.0), allowing for free access, distribution, and commercial use with proper attribution.

**Comment 6**

We note that Figure S1 includes an image of a participant in the study.

As per the PLOS ONE policy (http://journals.plos.org/plosone/s/submission-guidelines#loc-human-subjects-research) on papers that include identifying, or potentially identifying, information, the individual(s) or parent(s)/guardian(s) must be informed of the terms of the PLOS open-access (CC-BY) license and provide specific permission for publication of these details under the terms of this license. Please download the Consent Form for Publication in a PLOS Journal (http://journals.plos.org/plosone/s/file?id=8ce6/plos-consent-form-english.pdf). The signed consent form should not be submitted with the manuscript, but should be securely filed in the individual's case notes. Please amend the methods section and ethics statement of the manuscript to explicitly state that the patient/participant has provided consent for publication: “The individual in this manuscript has given written informed consent (as outlined in PLOS consent form) to publish these case details”.

**Response**

We have removed Figure S1 from the manuscript to comply with PLOS ONE’s policy on human subject’s research and protection of participant identity. No identifiable participant images remain in the revised version.

**Comment 7**

Please include captions for your Supporting Information files at the end of your manuscript, and update any in-text citations to match accordingly. Please see our Supporting Information guidelines for more information: <http://journals.plos.org/plosone/s/supporting-information>.

**Response**

Captions for all Supporting Information files (Figures S1–S3) have been added at the end of the manuscript, following the References section (line 601-608).

**Comment 8**

If the reviewer comments include a recommendation to cite specific previously published works, please review and evaluate these publications to determine whether they are relevant and should be cited. There is no requirement to cite these works unless the editor has indicated otherwise.

**Response**

The reviewers did not suggest any specific publications to be added to the manuscript

**9. Comment**

Please confirm at this time whether or not your submission contains all raw data required to replicate the results of your study.

**Response**

We confirm that the minimal data set required to replicate the results of this study is included within the Supporting Information files (S1 and S2 Data), which contain the raw species and genotype records by seasons, mosquito groups and geography, the specific values used to build all manuscript figures, and the underlying data points for statistical measures; furthermore, the specific methodology for the points extracted from images and laboratory observations is integrated into the Methods section (lines 192–194), with the corresponding raw values provided in the uploaded datasets to ensure full transparency and reproducibility in accordance with PLOS ONE policies.

10. **Comment**

Please amend the title either on the online submission form or in your so that they are identical.

Response

We have amended the title on the online submission form to exactly match the title provided in the manuscript file (line 1-2).

**11. Comment**

We note that there were multiple uploaded figures and SI figures in your file inventory. Please kindly remove your old upload and only retain the most resent one.

Response

We have cleared the old uploads from the system and retained only the finalized versions of the figures and Supporting Information. The current file inventory now contains only the most recent files."

# Point by point response to reviewer 1

**Manuscript title: Species Composition, Deltamethrin Susceptibility, and kdr Mutations in Anopheles Mosquitoes across Two Transmission Seasons in Northwest Ethiopia**

**I.	Main comments**

**Comment 1**

The title of the manuscript needs to be reformulated to adhere the main objective.

**Response**

The title has been revised to better reflect the study’s main objective (line 1-2).

**Comment 2**

In the abstract section, methodology and results need to be clarified. As the strain used (field and lab strain) or only field strain. Number of mosquitoes sample used for PCR. They were sampling from the exposed to deltamethrin mosquitoes or what? Idem for results.

**Response**

The abstract has been revised to clearly specify the mosquito populations used (field-derived only), the number and categories of samples analyzed by PCR, and the distinction between exposed and unexposed groups. Results have also been clarified accordingly (line 22-28).

**II. Minor comments**

**Introduction section:**

**Comment 1**

Line 61: can survive exposure to WHO diagnostic dose

**Response**

We have revised the paragraph to explicitly state that resistant mosquitoes can survive exposure to the WHO diagnostic dose, as suggested (line 65-66).

**Comment 2**

Line 85-86: “This study seeks to provide comprehensive assessment of Anopheles mosquito populations in Maksegnit and Gendawuha towns of Northwest 86 Ethiopia.” Please rephrase to take into account all the main words of the tittle.

**Response**

Thank you for this helpful suggestion. We agree that this sentence should better reflect the key terms and focus presented in the title. Consequently, the sentence has been rephrased to explicitly include the study’s core elements: species composition, deltamethrin susceptibility, *kdr* mutations, and transmission seasons (line 87-89).

**Comment 3**

Line 87: seasonal and geographical variation of what?

**Response**

We have clarified that the seasonal and geographical variations mentioned refer specifically to Anopheles mosquito populations (line 90).

**Methods and materials**

**Comment 1**

Line 106: Mosquito larvae, pupa collection, rearing, and adult collection, please specified clearly that mosquitoes were collected during the two seasons (rainy and after rainy)

**Response**

We agree that the sampling period should be clearly stated. Accordingly, we have revised the sentence to explicitly indicate that mosquito collection was conducted during both the rainy and post-rainy seasons (line 123).

**Comment 2**

Line 110: replace grown by reared

**Response**

We agree with the reviewer and have revised the term accordingly to ensure appropriate scientific terminology (line 126).

**Comment 3**

Line 111-112: reformulate the phrase to make easy to understand. Please give the quantity of Tetramin fish per tray and approximatively the number of larvae per tray.

**Response**

The rearing conditions have been clarified by specifying the approximate number of larvae per tray and the daily amount of TetraMin fish food provided (line 127-129).

**Comment 4**

Line 112: rephrase “Pupae were transferred into adult emergence cages and allowed to emerge in to adults” by Pupae were collected each morning with…. and transferred into adult emergence cages to allow emerge into adults

**Response**

Rephrased as suggested for clarity (line 130-131)

**Comment 5**

Line 121: rephrase “Twenty-five 3–5-day-old female Anopheles mosquitoes” into Twenty-five 3–5-day-old female Anopheles mosquitoes from field larvae collection,

**Response**

Corrected as suggested (line 139-140)

**Comment 6**

Line 122: deltamethrin-impregnated papers at what concentration?

**Response**

Thank you for this important observation. We agree that the concentration of deltamethrin should be clearly specified for reproducibility. Accordingly, we have revised the manuscript to include the concentration used in the bioassay (0.05%) (line 140).

**Comment 7**

Line 123: Control groups were left unexposed: control group must be exposed to untreated paper in the same condition as treated

**Response**

The text has been corrected to indicate that the control groups were exposed to untreated papers under the same experimental conditions as the test groups (line 141).

**Comment 8**

Line 123: After a one-hour exposure replace by After one-hour of exposure

**Response**

Corrected as suggested (line 142)

**Comment 9**

Line 125: When control mortality was between 5% and 20%, results were corrected using Abbott's formula. It was the case? If yes rephrase by treated mortality where corrected using Abbott's formula as control mortality was between 5% and 20%

**Response**

Corrected as suggested (line 144-145)

**Comment 10**

Line 129: 90–129 97% mortality indicates a resistance candidate please check and rephrase

**Response**

We carefully reviewed the statement and confirm that the original wording is correct as presented in the manuscript. Therefore, no modification has been made to this sentence (line 148).

**Comment 11**

Line 131: DNA extraction and species identification, please give the equipment and serial number used here

**Response**

Thank you for this valuable comment. We have revised the manuscript to include detailed specifications of the laboratory equipment used for DNA extraction and species identification. The names of the instruments, along with their manufacturers and model, have now been provided in the Methods section (line 155, 176-177).

**Comment 12**

Line 157: Detection of the L1014F and L1014S kdr alleles, please give the equipment and serial number used here

**Response**

The details of the PCR thermocycler, electrophoresis unit, and gel documentation system have been added as requested (line 186-187,189 and 190-191).

**Results**

**Comment 1**

Line 175: correct result by results

**Response**

We have corrected the typographical error as suggested (line 203).

**Comment 2**

Line 177: and replace by in the

**Response**

We agree with the reviewer and have revised the wording accordingly to improve grammatical accuracy.

**Comment 3**

Line 180: No seasonal variation was observed in the distribution of species; please give the statical result as p-value

**Response**

We have revised the sentence to include the corresponding p-value supporting the absence of seasonal variation in species distribution (line 210-211).

**Comment 4**

Line 182: groups (Fig. 2B-D), it shows two groups of mosquitoes and in line 177 you give only adult collection number. What about the exposure to deltamethrin samples used?

**Response**

The mosquitoes presented in Fig. 2B–D represent two experimental groups: deltamethrin-exposed and unexposed (control) adult mosquitoes. We have revised the manuscript to clearly indicate the sample allocation and exposure status, and to ensure consistency with the adult mosquito collection numbers reported in Line 177.

**Comment 5**

Line 190: delete during

**Response**

Corrected as suggested (line 226)

**Comment 6**

Line 193: can you review the table tittle?

**Response**

We have carefully reviewed and revised the table title to improve its clarity, accuracy, and alignment with the content presented in the table (line 229-230).

**Comment 7**

Line 201 – 204: can you give the number of each sub-group?

**Response**

The number of individuals in each sub-group has been added to the revised manuscript in (lines 237, 240–241).

**Comment 8**

Line 218: please be consistent with the expression. Used phenotypically susceptible instead of susceptible. Harmonize in the test.

**Response**

The term “phenotypically susceptible” is now used uniformly in place of “susceptible,” and the wording has been harmonized across all sections of the text.

**Figures section**

**Comment 1**

Fig 2: (A) shows the frequency of mosquito species (B) the species composition frequency by season and site for field collected adult (C), laboratory-reared susceptible (D), and laboratory reared resistant mosquitoes. Replace by Fig 2: (A) Prevalence of mosquito species (B) Species composition frequency by season and site for field collected adult (C) phenotypical susceptible mosquitoes after exposure to deltamethrin species frequency (D) phenotypical resistant mosquitoes after exposure to deltamethrin species frequency.

**Response**

Thank you for this constructive suggestion. We agree that the figure caption required clearer and more precise wording to better reflect the experimental groups and improve readability. We have revised the caption accordingly.

**Comment 2**

Fig 3: develop the tittle to allow well comprehensive and also the legend

**Response**

We have revised the title of Fig. 3 to make it more comprehensive and reflective of the figure content. However, the original figure legend has been retained as it already clearly describes the presented information.
