# Supplementary material for: Seasonal variation in species composition, deltamethrin susceptibility, and kdr mutations in anopheles mosquitoes in Northwest Ethiopia
Source: PLoS One. 2026 Jun 5;21(6):e0350942. doi: 10.1371/journal.pone.0350942 (PMC13240861; doi:10.1371/journal.pone.0350942)
Supplement: Attachment — Submitted filename: reviewer comments.docx [file pone.0350942.s007.docx]

**reviewer comments**

**Manuscript title:** Species Composition, Deltamethrin Susceptibility, and kdr Mutations in Anopheles Mosquitoes across Two Transmission Seasons in Northwest Ethiopia

I. **Main comments**
1. The title of the manuscript needs to be reformulated to adhere the main objective.
2. In the abstract section, methodology and results need to be clarified. As the strain used (field and lab strain) or only field strain. Number of mosquitoes sample used for PCR. They were sampling from the exposed to deltamethrin mosquitoes or what? Idem for results.

**II. Minor comments**

**Introduction section:**

Line 61: can survive exposure to WHO diagnostic dose

Line 85-86: “This study seeks to provide comprehensive assessment of Anopheles mosquito populations in Maksegnit and Gendawuha towns of Northwest 86 Ethiopia.” Please rephrase to take into account all the main words of the tittle.

Line 87: seasonal and geographical variation of what?

**Methods and materials**

Line 106: **Mosquito larvae, pupa collection, rearing, and adult collection,** please specified clearly that mosquitoes were collected during the two saisons (rainy and after rainy)

Line 110: replace grown by reared

Line 111-112: reformulate the phrase to make easy to understand. Please give the quantity of Tetramin fish per tray and approximatively the number of larvae per tray.

Line 112: rephrase “Pupae were transferred into adult emergence cages and allowed to emerge into adults” by Pupae were collected each morning with…. and transferred into adult emergence cages to allow emerge into adults

Line 121: rephrase “Twenty-five 3–5-day-old female Anopheles mosquitoes” into Twenty-five 3–5-day-old female Anopheles mosquitoes from field larvae collection,

Line 122: deltamethrin-impregnated papers at what concentration?

Line 123: Control groups were left unexposed: control group must be exposed to untreated paper in the same condition as treated

Line 123: After a one-hour exposure replace by After one-hour of exposure

Line 125: When control mortality was between 5% and 20%, results were corrected using Abbott's formula. It was the case? If yes rephrase by treated mortality where corrected using Abbott's formula as control mortality was between 5% and 20%

Line 129: 90–129 97% mortality indicates a resistance candidate please check and rephrase

Line 131: **DNA extraction and species identification,** please give the equipment and serial number used here

Line 157: **Detection of the L1014F and L1014S *kdr* alleles,** please give the equipment and serial number used here

**Results**

Line 175: correct result by results

Line 177: and replace by in the

Line 180: No seasonal variation was observed in the distribution of species, please give the statical result as p-value

Line 182: groups (Fig. 2B-D), it shows two groups of mosquitoes and in line 177 you give only adult collection number. What about the exposure to deltamethrin samples used?

Line 190: delate during

Line 193: can you review the table tittle?

Line 201 – 204: can you give the number of each sub-group?

Line 218: please be consistent with the expression. Used phenotypically susceptible instead of susceptible. Harmonize in the test.

**Figures section**

**Fig 2: (A)** shows the frequency of mosquito species **(B)** the species composition frequency by

season and site for field collected adult **(C),** laboratory-reared susceptible **(D),** and laboratoryreared

resistant mosquitoes. Replace by **Fig 2: (A)** Prevalence of mosquito species **(B) S**pecies composition frequency by season and site for field collected adult **(C)** phenotypical susceptible mosquitoes after exposure to deltamethrin species frequency **(D)** phenotypical resistant mosquitoes after exposure to deltamethrin species frequency.

**Fig 3:** develop the tittle to allow well comprehensive and also the legend
